# Efficacy and Safety of Laser Acupuncture in the Treatment of Children With Autism Spectrum Disorder: A Systematic Review

**DOI:** 10.1177/27536130261487432

**Published:** 2026-09-09

**Authors:** Deborah Dong, Lujie Xu, Sandy Thompson-Hodgetts, Hsing Jou, Sunita Vohra

**Affiliations:** 1 Department of Paediatrics, Faculty of Medicine and Dentistry, 3158 University of Alberta, Edmonton, Alberta, Canada; 2 Department of Psychiatry, Faculty of Medicine and Dentistry, Edmonton, Alberta, Canada; 3 Department of Occupational Therapy, Faculty of Rehabilitation Medicine, 3158 University of Alberta, Edmonton, Alberta, Canada

**Keywords:** laser acupuncture, children, autism, systematic review

## Abstract

**Background:**

Preliminary evidence suggests acupuncture may be helpful for pediatric autism; however, needles may be a source of healthcare trauma, so non-needle options should be prioritized.

**Objectives:**

To conduct a systematic review (SR) to synthesize the current evidence on the efficacy and safety of laser acupuncture for pediatric autism.

**Methods:**

We searched the following databases from inception until Nov 14, 2024: MEDLINE, CINAHL, EMBASE, Scopus, PsycINFO, Cochrane Library, SinoMed and China National Knowledge Infrastructure and J-Stage. Randomised controlled trials of laser acupuncture on children up to age 18 years of age with ASD were included. Outcomes were autism symptoms, adverse effects and language development indicators. The PRISMA-A checklist was used to guide conduct and reporting.

**Results:**

A total of two trials (n=76) were included. The quality of evidence for most indicators were considered low to very low by GRADE criteria. Meta-analysis was not possible due to heterogeneity in populations and outcomes. Results suggest acupuncture groups benefited compared to no treatment. There were no adverse effects reported by parents of the participants in either trial.

**Conclusions:**

Two studies indicate benefits of laser acupuncture in children with autism but given the low quality of evidence, results should be regarded with caution. Additional well conducted randomized controlled trials with larger sample sizes are required to build evidence.

**Protocol Registration:**

This protocol is registered in the International Prospective Register of Systematic Reviews (PROSPERO), registration number: CRD42024576887.

## Introduction

Autism Spectrum Disorder (ASD or autism) is a lifelong neurodevelopmental condition characterised by persistent difficulties in multiple contexts: (1) social communication and social interaction and (2) restrictive, repetitive behaviours, interests and activities (e.g., echolalia, idiosyncratic phrases).^1^ Language delays are often associated with autism.^2,3^ The prevalence of autism has been rising sharply, more than doubling to 1 in 36 children since 2008.^4^ Depending on the severity of core autism symptoms and associated symptoms/functional deficits, daily function can be impacted and can benefit from early and effective interventions.^5^

There are no medications at present that treat the core characteristics of autism.^6^ For psychological, behavioural or psychiatric symptoms, medications^7^ are associated with significant adverse events (AE) such as sedation, obesity, and tardive dyskinesia.^8^ The majority of therapeutic approaches aim at skill acquisition,^2,9^ however, they require extensive resources, time, effort, and funds.^10,11^ Complementary therapies are often used in chronic conditions, and parents of children with autism seek complementary treatments at high rates of 28% to 95%.^12^

Laser acupuncture (LA) uses a low-intensity non-thermal laser to stimulate acupuncture points.^13,14^ Low level laser irradiation has been shown to modulate gene expression, ATP production, reduce inflammation, and promote tissue generation and repair.^15-19^ A study of LA at the HT7 acupoint on Valproic Acid-Rat model of autism found decreased oxidative stress and neuroinflammation with improved cerebellar activity.^20^ An fMRI study on neurotypical adults found LA on the PC6 acupoint affected functional connectivity between brain areas.^21^ To date, LA has been found to benefit conditions such as musculoskeletal pain, headaches, postoperative nausea, and temporomandibular disorders.^22-25^ A systematic review (SR) on the safety of LA included 21 RCTs (n=1244) and concluded that it is safe, well tolerated, and associated with mild and transient adverse events (e.g., tingling, pain flare-up, transient fatigue), although more research and better reporting are required.^26^

Preliminary evidence from systematic reviews suggest needle acupuncture may be helpful for autism, including improved language abilities.^27-33^ However, sensory hyper-responsiveness^34,35^ and anxiety^36,37^ common to children with autism may potentially increase the fear of needles^38,39^ and make needle-based interventions challenging to implement. Although acupuncture and venipuncture needles are different, children and parents unfamiliar with acupuncture may think they are the same (and will feel the same fear/pain if they have a history of medical trauma). Laser acupuncture (LA) is a less invasive, painless option.^26,40^ A SR of the evidence of LA to assess its efficacy and safety for children with autism has not yet been done.

### Objectives

1. To synthesize evidence regarding the efficacy of LA on autism symptoms and/or associated functional challenges (e.g., speech and language delay, mood, self-care, cognitive or motor abilities,) of children with autism compared to controls.
2. To summarize adverse events associated with LA as reported in the included studies.

## Methods

### Protocol Registration

This protocol is registered in the International Prospective Register of Systematic Reviews (PROSPERO), ID# CRD42024576887.

### Types of Studies

RCTs, quasi-randomized controlled trials (e.g., trials using alternate allocation, allocation by birthdate) and controlled clinical trials that studied the efficacy of LA in children diagnosed with autism. No restrictions on language or publication date were applied.

### Types of Participants

All children under 18 years old diagnosed with autism spectrum disorder, including Asperger’s Syndrome, atypical autism and Pervasive Developmental Disorder-Not Otherwise Specified (PDD-NOS) were eligible. There were no restrictions to gender, race, or ethnicity. Diagnosis is according to the Diagnostic and Statistical Manual of Mental Disorders criteria.

### Types of Intervention

Eligible interventions included LA to acupoints standardised by the World Health Organisation (WHO). Studies that include LA in combination with other treatments were included. Laser treatments not specifically aimed at acupoints were excluded, as the purpose of this review is to evaluate the efficacy of lasers as a substitute for needle acupuncture.

### Types of Controls

All types of control were accepted, including the following: sham/placebo LA, no treatment, medication, wait list control, usual care, or active interventions other than LA. Sham LA refers to deliberate stimulation of non-acupuncture points using active laser. Placebo laser involves using a deactivated laser on a true acupuncture point. Both sham and placebo aim to mimic the treatment technique without causing activation of the physiological response to true LA.

### Types of Outcome Measures

#### Primary Outcomes

1. Severity of autism in children, measured by validated instruments, such as: Autism Diagnostic Observation Scale (ADOS), Childhood Autism Rating Scale (CARS), Autism Diagnostic Interview, Revised (ADI-R), Autism Treatment Evaluation Checklist (ATEC), Aberrant Behaviour Checklist (ABC).
2. Reports of adverse events associated with LA in the included studies.

#### Secondary Outcomes

Associated functional deficits (e.g., speech and language delay, daily functional skills, motor skills, mood, anxiety, emotional regulation), assessed using validated instruments, such as: Preschool Language Scales, Clinical Evaluation of Language Fundamentals - 5th ed. (CELF-5 or Preschool (CELF-P2), Expressive Vocabulary Test - 2nd Edition (EVT-2), Functional Independence Measure for Children (WeeFIM), Movement Assessment Battery for Children (Movement ABC), or other validated scales.

### Literature Search Strategy

The search process was conducted with the assistance of a health research librarian at the University of Alberta (ED). A comprehensive search of the following nine databases from inception to November 14, 2024 was conducted: MEDLINE, CINAHL, EMBASE, Scopus, PsycINFO, Cochrane Library, SinoMed, China National Knowledge Infrastructure and J-Stage. There were no limitations on publication date or language. For the English language databases, we used keywords related to LA (laser acupuncture, laserpuncture, laser therapy, low level laser therapy, cold laser) and Autism Spectrum Disorder (Autism, Aspergers, autism spectrum disorder, autistic, PDD-NOS). See supplementary Appendix 1 for full search strategies. For the Chinese databases, keywords were searched (e.g., zibizheng, guduzheng, jiguangzhenjiu). For the Japanese database, keywords were autism and laser acupuncture. Between July 25 - November 14, 2024, we searched clinicaltrials.gov, Proquest Dissertations and Theses, Google Scholar, the reference list of relevant studies, and emailed authors of relevant studies for information on additional studies. No language restriction was applied. The Preferred Reporting Items for Systematic Reviews and Meta-Analyses of Acupuncture flow chart recorded our decisions (PRISMA-A).

### Study Selection

All search results were downloaded into Covidence for study screening, selection and management, and duplicates removed (Covidence, 2025). Two reviewers (DD and LX) independently screened abstracts for all potentially relevant studies based on the inclusion/exclusion criteria. For those meeting inclusion criteria, both reviewers assessed eligibility of the full articles. All disagreements were resolved by discussion, and consensus was able to be reached without an arbiter.for Intervention Description and Replication Checklist (TIDier)^41^ and Standards for Reporting Interventions in Clinical Trials of Acupuncture (STRICTA)^42^ were used to evaluate report completeness.

### Data Extraction and Management

The data were extracted by one author (DD) and independently reviewed for accuracy by a second (LX). Data were extracted using a pre-designed, piloted, standardised data collection form stored in Excel. Details of data extracted is available in Appendix 2. The completed data extraction forms are provided in Appendix 3.

### Risk of Bias Assessment

DD and LX independently completed RoB assessments of the included studies using the Cochrane Risk of Bias (RoB 2) tool,^43^ and consulted the senior author (SV) to resolve discrepancies. Full consensus was reached. Following the Cochrane Handbook for Systematic Reviews of Interventions,^44,45^ all seven domains were evaluated and rated as low, unclear, or high RoB according to the algorithm. Each study was then given an overall rating of low, unclear, or high RoB.

#### GRADE Assessment

The certainty of evidence for each outcome was reviewed independently by DD and LX for the 5 domains of GRADE^46^: risk of bias, inconsistency, indirectness, imprecision and publication bias. All disagreements were resolved by consulting the senior author (SV). Full consensus was reached. Each outcome was rated as high, moderate, low or very low certainty of evidence.

### Synthesis and Evaluation of Data

We reported the mean difference for all continuous outcomes between baseline and follow up measures, with standard deviation and 95% confidence intervals. This allowed us to present the size of the intervention effect and the variability. AE reported in the included studies were summarized in narrative form. An adverse event is defined as any “unfavourable and unintended sign, symptom, or disease temporally associated with the use of a medical treatment or procedure that may or may not be considered related to the medical treatment or procedure. These may include discomfort, skin irritation or worsening of symptoms”.^47^

We attempted to contact the lead authors of both included studies for additional information. If a response was not received, our attempts to obtain data were noted. PRISMA for acupuncture (PRISMA-A)^48^ guided the reporting of this review.

## Results

### Search Results

**Figure 1.**
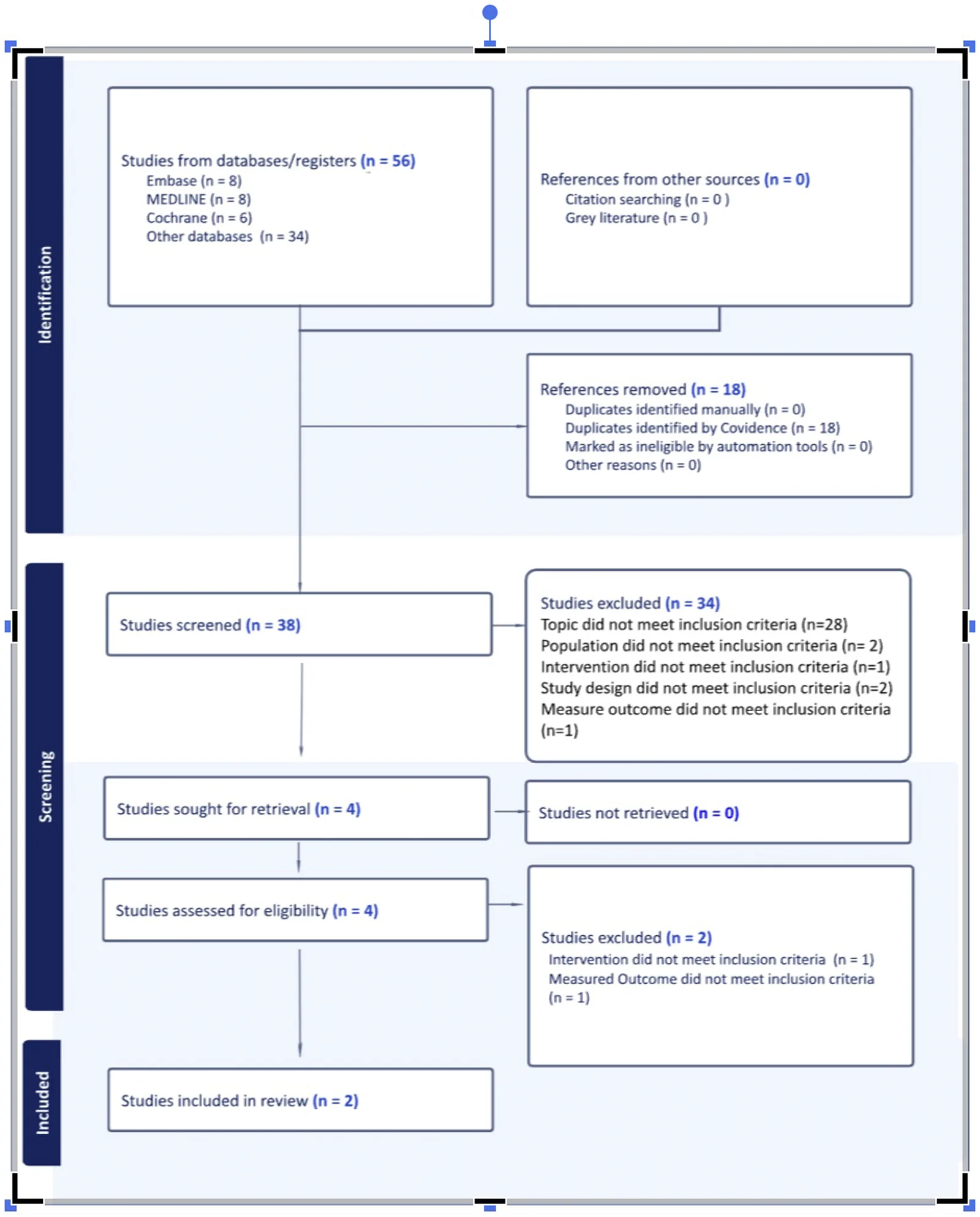
PRISMA flow diagram

The database search found 56 studies: 18 duplicates were automatically removed by Covidence, and the reviewers excluded an additional 34 abstracts after screening. The full texts of the remaining four studies were reviewed, and two of them were excluded. One study’s intervention did not meet inclusion criteria as it did not use low level laser therapy on acupuncture points.^49^ The other study was not randomized; it applied LA to two siblings, with effects measured by electroencephalography (EEG).^50^ This did not meet the outcome nor RCT design inclusion criteria. No records were identified through other sources. In the end, two trials were eligible for inclusion (Surapaty et al, 2020; Elsheikh et al, 2023).^51,52^ See Figure 1 for the PRISMA flow diagram.

### Study Characteristics of Included Trials

Two trials met inclusion criteria with a total of 76 participants. There was equal distribution of experimental and control groups. For both RCTs, baseline data were gathered prior to the start of the study and follow-up data were gathered at the end of six weeks. The CONSORT 2010 statement advises against testing for baseline differences,^53^ however, both studies conducted statistical testing for baseline differences and are therefore summarized in this review. There were no significant age, gender or language differences between intervention and control groups reported at baseline. Both studies were conducted in outpatient clinics and reported no dropouts. In terms of study design, both are considered RCTs. Surapaty et al (2020) was a double-blind parallel group trial.^51^ Although Elsheikh’s study was described as an observational longitudinal study, participants were randomized into intervention and control arms. Study authors did not respond to our request for clarification and we included this study as an RCT.^52^

See characteristics of included studies in Table 1. Surapaty et al.’s study, conducted in Indonesia, included 46 children between the ages 2 - 6 years. All children were confirmed to have autism by a pediatric neurologist. Specifics of diagnostic tools or criteria were not provided. Children were excluded from the study if they used medications (e.g., psychotropic drugs, high-dose corticosteroids, nonsteroidal immunosuppressants), had contraindications to LA (defined as malignancy, uncontrolled epilepsy, infections with body temperatures ≥38°C, wounds, or dermatitis in the acupuncture-point areas), medical emergencies, increased photoallergic responses, cytostatic therapy, acute exacerbations of chronic skin disorders (e.g., acute dermatitis, systemic lupus erythematosus, or cutaneous tuberculosis), and patients who were uncooperative.^51^

**Table 1.** Characteristics of Analyzed Trials

Lead author	Country	Year of Publi-cat--ion	Study design	Sample size (I/C)	Mean age (I/C)	Gender (%male) (I/C)	Methods of Interven-tion	Control	Co-Interven-tion	Outcomes measures	Safety
Surapaty	Indonesia	2020	RCT	23/23	3/3	95.7/78.3	LA + SI	Placebo (inactive laser to true acupunct-ure points) + SI	SI	WeeFIM (language comprehension, expression, social interaction) Sensory Profile: parental report	Child given goggles to wear
Elsheikh	Egypt	2023	RCT	15/15	7.8±2.8/8.5±2.7	73.7/66.7	LA + Parents provided a rich language environ-ment	No LA + Parents provided a rich language environ-ment	Parents provided a rich language environment	CARS, Arabic Preschool Language Scale (receptive, expressive, and total language)	Child wore protective eyewear

CARS, Childhood Autism Rating Scale; I/C, Intervention/Control; WeeFIM, Functional Independence Measure for Children; LA, laser acupuncture; SI, Sensory Integration therapy.

Elsheikh et al.’s trial, conducted in Egypt, included 30 children between the ages 5-12 years diagnosed with autism according to Diagnostic and Statistical Manual of Mental Disorders Fifth Edition (DSM-5) and Autism Diagnostic Interview-Revised (ADI-R) criteria. Children were excluded from the study in cases of: abnormal neurological examination, comorbid neuropsychiatric disorders, delayed motor development, and manifestations suggestive of syndromic involvement, receiving drugs, history of developmental delay, or current or past phoniatric therapy (the medical or therapeutic treatment of organs involved in speech production).^52^

### Characteristics of Interventions

Both trials were six weeks long. Treatment was based on the Traditional Chinese Medicine (TCM) principles as opposed to different styles such as Western medical or Japanese acupuncture. There was no individualisation of treatment reported. Each study involved LA treatment to the intervention groups; the control groups received placebo acupuncture in one study (Surapty et al) or no treatment in the second study (Elsheikh et al). There are no details regarding the type, training or experience of the practitioners delivering the LA treatments for either study. Both studies utilized LA combined with another therapy in both the intervention and control groups. Surapaty et al utilized sensory integration and Elsheikh et al counseled families to provide a rich language environment in the home. No details of the specific exercises, frequency, duration, rationale, or providers of these other therapies were reported.

The Surapaty et al study used an RJ-Laser laserpen set at Nogier G 18688-Hz frequency modulation wave, 40 mW power, 785 nm (infrared wavelength) and delivering 1 Joule of energy per acupoint in 25 seconds. Treatment was three times a week for a total of 18 sessions. The Elsheikh et al study used a Gallium aluminum arsenide laser, 850 nm (infrared wavelength), 200mW delivering 3 Joules/c㎡ per acupoint; frequency setting was unclear. Treatment was two times a week for a total of 12 sessions.

Five acupuncture points were used in both studies: GV20 (Baihui), bilateral LI4 (Hegu), bilateral SP6 (Sanyinjiao), bilateral ST36 (Zusanli), and bilateral speech area (scalp acupuncture). Surapaty et al also used the points bilateral HT7 (Shenmen), bilateral GB34 (Yanglingquan) and bilateral LR3 (Taichong). Elsheikh et al used the points GV24 (Shenting), GV26 (Shuigou), bilateral GB20 (Fengchi), and EXHN1 (Sishencong). See characteristics of LA treatment summarized in Table 2.

**Table 2.** Details of Laser Acupuncture Treatment

Lead author	Individual-ised?	Acupuncture points	Laser model	Power (mW)	Time per point (seconds)	Frequency (Hz)	Dosage (J/cm^2^ per acupuncture point)	Wave-length (nm)	Total Number of treatments	Frequency of treatment	Duration of treatment
Surpaty	No	GB34, LR3, HT7, LI4, SP6, ST36, GV20, Scalp-Speech Area	RJ-Laser Laserpen Praxis type130	40	25	Nogier G 18688	1	785	18	3x/week	6 weeks
Elsheikh	No	GB20, LI4, SP6, ST36, GV20, GV24, GV26, Ex-HN1, Ex-HN3, Scalp-Speech Area	Gallium aluminum arsenide laser	200	12	Not specified	3	850	12	2x/week	6 weeks

### Risk of Bias Assessment

**Figure 2.**
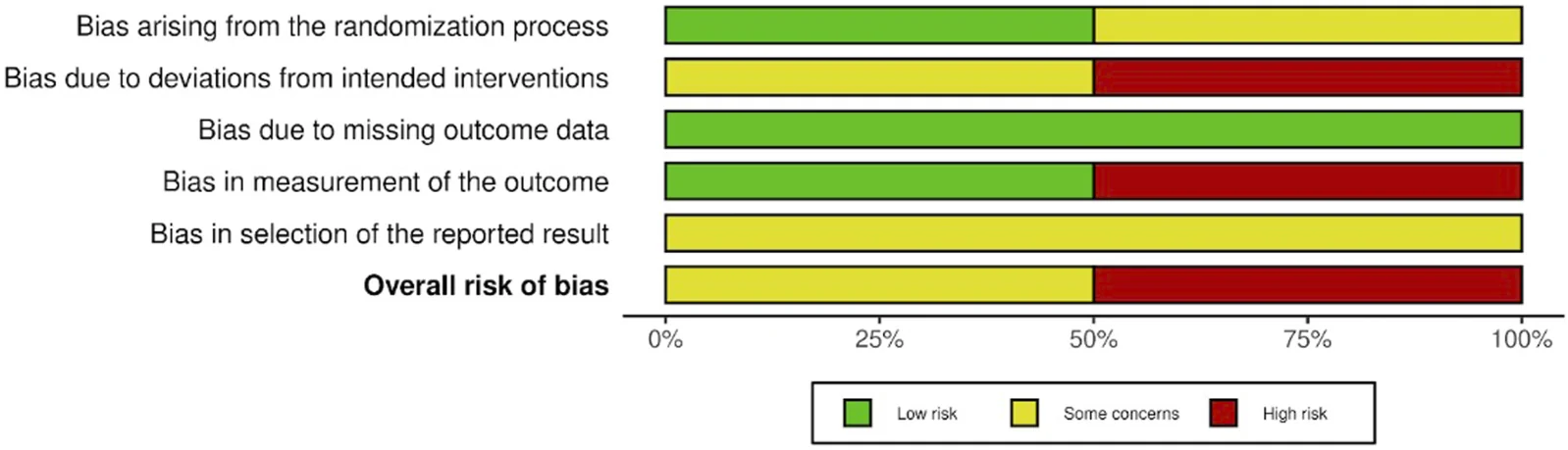
Risk of bias graph

**Figure 3.**
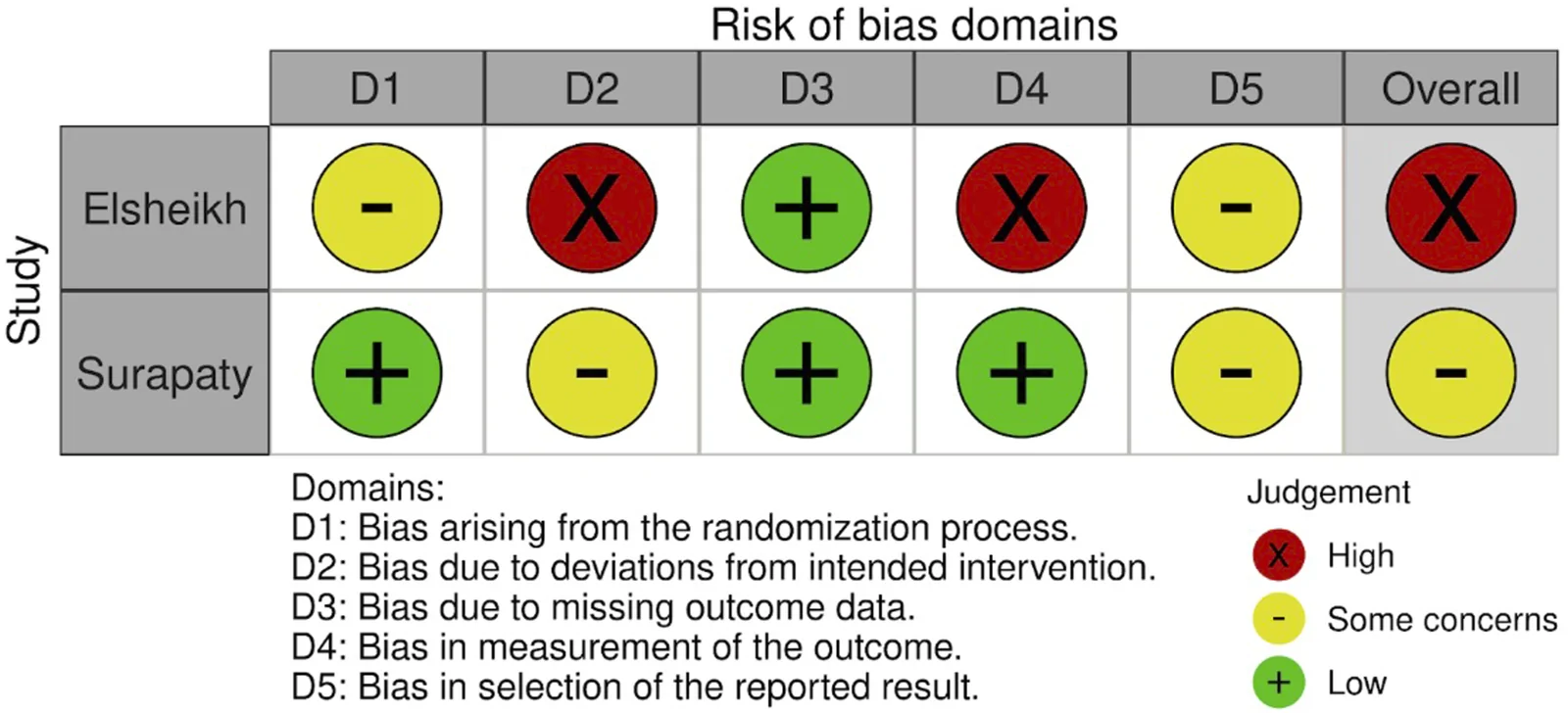
Risk of bias summary

RoB was conducted across five domains using the Cochrane Risk of Bias 2 tool.^43^ The Surapaty et al study is rated overall as having ‘some concerns’ of risk of bias. The Elsheikh et al study is rated as having an overall ‘high’ risk of bias. Figure 2 provides a graph of the RoB assessment over both studies. Figure 3 provides a summary of each item for each included study.

### GRADE Assessment

Based on the GRADE criteria, the quality of evidence for autism severity (measured by CARS), receptive language, expressive language and total language score (all measured by the Arabic Preschool Language Scale (APLS)) were downgraded to very low certainty due to imprecision (only one study) and overall high RoB of the study. The quality of evidence for language comprehension, language expression, and social interaction (measured by the WeeFIM) were downgraded to low certainty due to imprecision (only one study). See Table 3 for a summary of the GRADE assessment.

**Table 3.** GRADE Assessment

Outcome	No. of studies/No. Of partici-pants	Intervention/Control	Certainty of the evidence (GRADE)					Certainty
			Risk of bias	Inconsistency	Indirectness	Imprecision	Other considerations	
Autism Severity (1)	1/30	15/15	Very serious	Not serious	Not serious	Very serious	Large Effect	Very low
Receptive Language (2)	1/30	15/15	Very serious	Not serious	Not serious	Very serious	None	Very low
Expressive Language (2)	1/30	15/15	Very serious	Not serious	Not serious	Very serious	None	Very low
Total Language Score (2)	1/30	15/15	Very serious	Not serious	Not serious	Very serious	Large effect	Very low
Language Comprehens-ion (3)	1/46	23/23	Not serious	Not serious	Not serious	Very serious	Large effect	Low
Language Expression (3)	1/46	23/23	Not serious	Not serious	Not serious	Very serious	​	​
Social Interaction (3)	1/46	Not serious	Not serious	Not serious	Not serious	Very serious	​	​

(1) Measured using the Childhood Autism Rating Scale; (2) Measured using the Arabic Preschool Language Scale; (3) Measured using the Functional Independence Measure for Children; CI = Confidence Interval; MD=Mean Difference.

### Outcome Measures of Interest

Meta-analysis was not completed as there was significant heterogeneity between the studies’ populations, measured outcomes and instruments used to measure outcomes. While both trials assessed expressive language, the difference in participant age groups (2-6 years, and 5-11 years) prevented meta-analysis. Additional subgroup and sensitivity analyses were not possible due to limited data. A funnel plot to assess potential publication bias was not possible due to insufficient number of studies.

Elsheikh et al measured the overall severity of autism using the validated Childhood Autism Rating Scale (CARS)^54^ and language skills using the APLS, reported by the author to be standardized (N.H. Nashaat, email communication, Sept 17, 2024). Surapaty et al used the language components of the WeeFIM.^55^

### Primary Outcomes

1. Autism severity

Elsheikh et al reported improved (lower) CARS scores, indicating decreased autism severity in the LA group compared to no treatment (MD -3.4, 95% CI -5.5 to -1.4; n=30).

2. Adverse Effects

Both studies asked the parents of the participants about adverse effects, and none were reported. Neither study employed a structured method (e.g., checklist of types of adverse effects, when it was asked) to collect this data.

### Secondary Outcomes

1. Receptive Language

Elsheikh et al, using the APLS, reported improved (higher) receptive language scores in the LA group compared to no treatment (MD 0.9, 95% CI 0.1 to 1.8; n=30).

2. Expressive Language

Elsheikh et al, using the APLS, reported improved (higher) expressive language scores in the LA group compared to no treatment (MD 1.0, 95% CI 0.1 to 1.9; n=30). Surapaty et al, using the WeeFIM, reported improved (higher) expressive language scores in the LA group versus placebo (MD 1.04, 95% CI 0.84 to 1.24; n=46).

3. Total language score

Elsheikh et al, using the APLS, reported improved (higher) total language scores in the LA group compared to no treatment (MD 2.1, 95% CI 0.6 to 3.5).

4. Language Comprehension

Surapaty et al, using the WeeFIM, reported improved (higher) language comprehension scores in the LA group versus placebo (MD 1.91, 95% CI 1.69-2.13).

6. Social Interaction

Surapaty et al, using the WeeFIM, reported improved (higher) social interaction scores in the LA group versus placebo (MD 1.47, 95% CI 1.19 to 1.75).

Table 4 provides a summary of effects.

**Table 4.** Summary of Effects

Elsheikh: No. of participants: n=30					
​	Intervention (MD pre and post)	Control (MD pre and post)	Relative effect (mean ± SE)	95% confidence interval	p-value
Autism severity (CARS) (lower represents improvement)	-5.6 ± 3.4	-2.1 ± 1.7	-3.4 ± 1.0	-5.5 to -1.4	0.002
Receptive language raw score (APLS) (higher represents improvement)	1.6 ± 1.4	0.7 ± 0.7	0.9 ± 0.4	0.1 to 1.8	0.036
Expressive language raw score (APLS) (higher represents improvement)	1.7 ± 1.5	0.7 ± 0.8	1.0 ± 0.4	0.1 to 1.9	0.033
Total language raw score (APLS) (higher represents improvement)	3.3 ± 2.4	1.3 ± 1.2	2.1 ± 0.7	0.6 to 3.5	0.007

Surapaty: No. of participants: n=46					
​	Intervention (MD pre and post)	Control (MD pre and post)	Relative Effect (Mean ± SE)	95% Confidence Interval	p-value
Language comprehension (WeeFIM) (higher represents improvement)	2.86 ± 0.86	0.95 ± 0.63	1.91	1.69 to 2.13	< 0.005
Expressive language (WeeFIM) (higher represents improvement)	1.73 ± 0.81	0.69 ± 0.55	1.04	0.84 to 1.24	< 0.005
Social interaction (WeeFIM) (higher represents improvement)	2.86 ± 1.09	1.39 ± 0.78	1.47	1.19 to 1.75	< 0.005

**MD:** mean difference; **SE:** Standard error; **LA:** laser acupuncture; **CARS**: Childhood Autism Rating Scale; **APLS:** Arabic Preschool Language Scale; **WeeFIM**: Functional Independence Measure for Children.

### Additional Outcomes

Surapaty et al utilised a Parental Report - Sensory Profile. There was no response to an email request about this tool, therefore it was not included in this review. Elsheikh et al additionally measured brain-derived neurotrophic factors levels and miR-320 expression but these results were not within the scope of this study.

## Discussion

### Summary of Main Results

The aim of this SR was to evaluate the efficacy and safety of LA on autism symptoms and/or associated functional deficits in children. Two studies with a total of 76 participants met inclusion criteria. Meta-analysis was not completed due to the small number of studies and heterogeneity between the studies. Comparison of test scores before and six weeks after treatment found that laser acupuncture reduced autism severity, and improved receptive language, language comprehension, expressive language and social interaction skills in intervention groups compared to controls. Both studies monitored adverse effects and none were reported by parents.

### Strength and Limitations of This Review

A strength of this systematic review is the comprehensive search of databases, registries, and grey literature. The methodology adhered to the rigorous standards of the Cochrane Handbook of Systematic Reviews, TIDieR, and GRADE, and our reporting standards are according to the PRISMA-A checklist.^48^ The checklist is provided in Appendix 4.

There are a number of limitations. Publication bias and false positive (Type 1) error are significant issues in small studies,^56,57^ therefore the results of these included studies should be treated with caution. Secondly, the small number of studies and heterogeneity of measured outcomes precluded meta-analysis. Thirdly, both studies lack protocol registration so assessing protocol deviations and outcomes reporting bias were not possible. Importantly, one study was determined to have high RoB due to lack of blinding, and the other also had some concerns of RoB. This, and the low number of studies, led to the results being downgraded to low or very low certainty (GRADE). Both studies were of short duration (six weeks), so the long-term effects of LA cannot be determined, an important consideration for a lifelong condition such as autism. Both studies involved different co-interventions (sensory integration in Surapaty et al.’s study, and rich language environment in Elsheikh et al.’s study) and it is difficult to assess their impact due to lack of sufficient details. The reports of both studies were missing important details such as whether there were any deviations from the intervention or protocol as intended. We contacted the authors and received one email response from Elsheikh et al study’s contact author but no further correspondence ensued. Surapaty et al did not respond to requests for information.

An additional limitation is lack of generalizability to other populations given differences in culture, resources, and provision of therapeutic services compared with those found in Egypt and Indonesia. The study by Elsheikh et al employed restrictive inclusion criteria, excluding children with abnormal neurological examination, comorbid neuropsychiatric disorders, and/or delayed motor development, thus potentially overlooking more severely impacted children and affecting generalizability. Finally, although no AEs were identified in either study, it is known that the reporting and analysis of adverse events in RCTs is often poor,^58^ therefore the lack of reported adverse events in the context of this review should be regarded with caution.

### Consensus and Divergence From Other Literature

To our knowledge, this is the first SR to assess the efficacy and safety of LA on children with autism. Previous SRs have evaluated needle acupuncture for children with autism.^27-32,59,60^ The two earliest SRs concluded there was inadequate evidence to support the use of acupuncture.^59.60^ In comparison, the most recent SRs showed preliminary evidence of benefit, although the quality of evidence was low or very low due to issues of poor study methodology, small studies and high RoB.^27-32^ Laser and needle acupuncture are based on the same treatment principles of stimulating selected acupuncture points to achieve therapeutic effects. However, the mechanism differs, with laser relying on the principles of photo-biomodulation,^61-67^ whereas needles provide mechanical stimulation.^68-73^

Our study also found benefits from the stimulation of acupuncture points. Yang et al (2020), in a SR evaluating the safety of LA, concluded that LA appears to be safe and is associated with a few mild and transient adverse events: tingling, pain flare-ups, and fatigue.^26^ Our study supports evidence of safety of LA: no adverse events were reported in our included studies.

## Implications for Practice

Our SR found LA was associated with decreased autism severity and improved language and social interaction skills but the findings were based on only two small studies with high risk of bias and low to very low quality evidence. Consequently, evidence is insufficient to recommend either for or against the use of laser acupuncture in children with autism at this time. Nonetheless, it is important to acknowledge that many families are seeking options to supplement conventional therapeutic approaches. Compared to needle acupuncture, which is supported by a relatively larger body of research regarding potential benefits for children with autism, LA may offer advantages due to its non-invasive application. Furthermore, this and other SRs have identified it is a modality associated with low adverse effects, suggesting a safe option for families to investigate.

## Implications for Future Research

Rigorous RCTs with larger sample sizes are required to determine the efficacy and safety of LA for children with autism. A challenge of this SR was the lack of sufficient high quality data from which to draw conclusions. The International Consortium for Health Outcomes Measurement (ICHOM) promotes a set of patient-centred outcome measures for autism which may streamline the measurement of outcomes and increase comparability of studies.^74^ Our included studies used diverse treatment parameters (e.g., power of laser, dosage, frequency and acupuncture points) and standardization of these would also improve the comparability of outcomes. Trials complying with the Standards of Reporting Interventions in Clinical Trials of Acupuncture (STRICTA) is recommended. Both studies included concomitant therapy, in addition to laser acupuncture. However, details of the concomitant therapy provided, treatment frequency and duration, session length, qualifications of the therapy providers were not reported. In future studies, standardized reporting for concomitant interventions are necessary to ensure clarity in evaluating intervention efficacy. The study by Surapaty et al demonstrated that blinding of participants and clinicians in laser acupuncture trials is possible, a barrier that has proven more challenging with needle acupuncture.

Studies on the efficacy of laser acupuncture for functional issues associated with autism, like mood, sleep, cognitive development, self-care, and motor skills were not available. Research in these areas is important to support the quality of life of this population.

The two studies measured harms through informal parent reporting. Future studies should systematically monitor adverse events using validated tools or checklists and adopt standardised reporting methods to improve the consistency and transparency of harm evaluation.

## Conclusion

Although both studies report large effects, all outcomes are low to very low certainty and should be regarded with caution. No adverse events were reported. There is inadequate evidence to determine the efficacy or safety of LA in the management of children with autism at this time. Rigorous RCTS with larger sample sizes and longer follow-up are required.

## Supplemental Material

Supplemental Material - Efficacy and Safety of Laser Acupuncture in the Treatment of Children With Autism Spectrum Disorder: A Systematic Review Supplemental Material for Efficacy and Safety of Laser Acupuncture in the Treatment of Children With Autism Spectrum Disorder: A Systematic Review by Deborah Dong, Lujie Xu, Sandy Thompson-Hodgetts, Hsing Jou, MD, Sunita Vohra in Global Advances in Integrative Medicine and Health

## Appendix

### Abbreviations

Autism Spectrum Disorder: ASD
systematic review: SR
randomized controlled trial: RCT
Traditional Chinese Medicine: TCM
Grading of Recommendations, Assessment, Development and Evaluation: GRADE
Risk of Bias 2 Tool: RoB 2
Preferred Reporting Items for Systematic Reviews and Meta-Analyses of Acupuncture: PRISMA-A
