# Supplementary material for: Efficacy and Safety of Laser Acupuncture in the Treatment of Children With Autism Spectrum Disorder: A Systematic Review
Source: Glob Adv Integr Med Health. 2026 Sep 9;15:27536130261487432. doi: 10.1177/27536130261487432 (PMC13558927; doi:10.1177/27536130261487432)
Supplement: Supplementary file 1 [file sj-pdf-1-gam-10.1177_27536130261487432.pdf]

Appendices

Appendix 1. Search Strategy

Medline

1.exp Child Development Disorders, Pervasive/

2. exp Autistic Disorder/

3. autis$.mp.

4. asd.ti,ab.

5. kanner$.ti,ab.

6. asperger$.ti,ab.

7. (pervasive and development and disorder).ti,ab.

8. PDD.mp. or PDD-nos.ti,ab.

9. childhood disintegrative disorder.ti,ab.

10. 1 or 2 or 3 or 4 or 5 or 6 or 7 or 8 or 9

11. (laser acupuncture or laser acutherapy).mp.

12. (low level light therapy or low level laser therapy or low level laser acupuncture or laserpuncture or LLLT or LLLA).mp.

13. cold laser.mp.

14. 11 or 12 or 13

15. 10 and 14

Scopus

"laser acupuncture*" OR "laserpuncture*" OR "laser therapy*" OR " low level laser therapy*" OR "cold laser*"

AND

"autism*" OR "Autism Spectrum Disorder*" OR "autistic*" OR "Asperger's*" OR "Pervasive Developmental Delay - Not Otherwise Specified*" OR "PDD-NOS*" OR "kanner syndrome*"

PsycINFO

1. exp Autism Spectrum Disorders/
2. autis$.mp.
3. asd.ti,ab.
4. kanner$.ti,ab.
5. asperger$.ti,ab.
6. (pervasive and development and disorder).ti,ab.
7. PDD.mp. or PDD-nos.ti,ab.
8. childhood disintegrative disorder.ti,ab.
9. 1 or 2 or 3 or 4 or 5 or 6 or 7 or 8
10. (laser acupuncture or laser acutherapy).mp.
11. (low level light therapy or low level laser therapy or low level laser acupuncture or laserpuncture or LLLT or LLLA).mp.
12. cold laser.mp.
13. 10 or 11 or 12
14. 9 and 13

Cochrane Library (Wiley interface)

(autis* or asd or kanner* or asperger* or (pervasive and development and disorder) or PDD or PDD-nos or "childhood disintegrative disorder") AND ("laser acupuncture" or "laser acutherapy" or "low level light therapy" or "low level laser therapy" or "low level laser acupuncture" or laserpuncture or LLLT or LLLA or "cold laser")

Proquest theses search

Search terms:

(autis* or asd or kanner* or asperger* or (pervasive and development and disorder) or PDD or PDD-nos or "childhood disintegrative disorder")

And

"laser acupuncture" or "laser acutherapy" or "low level light therapy" or "low level laser therapy" or "low level laser acupuncture" or laserpuncture or LLLT or LLLA or "cold laser"

Appendix 2. Data Extraction

1. Characteristics of included studies (title, lead author, journal, year of publication, setting, registration number, acupuncture paradigm)
2. Study methods (design, randomization, allocation concealment, blinding, funding)
3. Participants (inclusion/exclusion criteria, sample size, age and sex distribution, baseline measures regarding severity of autism, language)
4. Trial characteristics (acupuncture points, rationale, extent to which treatment was individualised, control intervention, number and frequency of treatment, duration of treatment, follow-up period)
5. Co-interventions (new or ongoing treatment, rationale, frequency)

6. Instructions (to practitioners, parents, informed consent)

6. Adverse events

7. Laser settings (type of laser used, power, time of irradiation, frequency, dosage, wavelength)

8. Practitioner background (type of practitioner, training, years of experience, other relevant information)

9. Follow-up (Number of drop-outs, reason for drop-out, analysis type)

10. Outcome data (Mean difference, standard deviation)

11. Other outcomes reported but not used in this review

12. References to other eligible trials, both published and unpublished

Appendix 3.

Surapaty et al. Data Extraction Form

Name of data extractors:	D Dong, L Xu		
Date of data extraction: DD/MM/YY	06/09/2024		
PROSPERO Study ID	CRD42024576887		
**Characteristics of included studies**			
Title	Laser Acupuncture Effects on Speech and Social Interaction in Patients with Autism Spectrum Disorder		
Lead Author (Last name, initials)	Surapaty, I.A.		
Journal	Medical Acupuncture		
Year Published in print (latest publication will be used if there is publication errata).	2020		
Country of first author	Indonesia		
Multicentre (Y/N)	Yes		
Number of Centres	3		
Setting(s)	outpatient clinic		
Trial registry ID			
Trial registration document/protocol available? If yes, append to study (URL, DOI, or PMID)			
Acupuncture paradigm (if described)	☐ TCM<br>☐ Western medical<br>☐ Japanese<br>☐ Korean<br>☐ Five Element<br>☐ Auricular<br>☐ Other<br>☑ Not specified		
**Study Methods**			
Study Design:	☑ RCT<br>☐ Quasi RCT<br>☐ crossover<br>☐ Controlled Clinical Trial		
	**Judgement**	**Support for Judgement (pg. number)**	
Was random sequence generation used	Yes	p 301	
What kind	random number table	p 301	
Was allocation concealment used	Yes	p 302	
Was there blinding of:			
a)participants	Yes	p 302	
b)treating clinician	Not reported		
c)outcome assessor	Yes	p 302	
d)statistician	Not reported		
e)data safety monitoring board	Not reported		
Source(s) of Funding	No funding	p.308	
**Participant Characteristics**			
Study's Participant Inclusion Criteria:	☑ Autism Diagnosis<br>☑ under 18 years old		
Study's Participant Exclusion Criteria	☐ psychiatric disorder<br>☐ other neurological condition<br>☐ neuropsychiatric disorder<br>☐ syndromic involvement<br>☐ Developmental Delay<br>☑ Psychotropic Drugs<br>☑ High Dose Corticosteroids<br>☐ medications<br>☑ Uncontrolled Epilepsy<br>☐ Medical emergency<br>☐ Increased photoallergic response<br>☐ Exacerbation to chronic skin disorders<br>☐ Phoniatric treatment<br>☐ Motor delay<br>☑ Malignancy<br>☑ Infections with fever<br>☑ increased photoallergic responses<br>☑ Uncooperative<br>☑ Other<br><br>*Check all that apply		
	**Intervention**	**Control**	**Significant difference? (Comparability of groups at baseline)**
Sample Size	23	23	
Age range	2-5 year	2-6 years	No
Mean Age ± S.D.	3	3	No
Male:Female percentage	95.7%: 4.3%	78.3%: 21.7%	No
Severity of autism (CARS score range)	not available	not available	
Speech scores (mean and range)			
comprehension	2	2	No
expression	2	2	No
Social interaction	2	2	No
Parental Report (Sensory Profile questionnaire)	70	65	No

Trial Characteristics			
**A. Details of Laser Acupuncture Intervention**			
Names (or location if no standard name) of acupuncture points used	Check all that apply<br>[x] GB34 (Yanglingquan) - bilateral<br>[x] LR 3 (Taichong) - bilateral<br>[x] HT7 (Shenmen) - bilateral<br>[x] LI4 (Hegu) - bilateral<br>[x] Sp6 (Sanyinjiao) - bilateral<br>[x] ST36 (Zusanli) - bilateral<br>[ ] GB20 (Fengchi) - bilateral<br>[x] GV20 (Baihui) - unilateral<br>[ ] GV24 (Shenting) - unilateral<br>[ ] GV26 (Shuigou) - unilateral<br>[ ] Ex-HN1 (Sishencong) - set of 4 points<br>[ ] Ex-HN3 (Yintang) - unilateral<br>[x] Scalp-Speech Area - a line on scalp<br>[ ] Other		
Rationale for points	[ ] based on previous studies<br>[ ] literature sources<br>[ ] Consensus methods<br>[ ] Other<br>[x] Not reported		
Extent to which treatment was individualised	[x] none (all patients receive the same treatment at all sessions)<br>[ ] partial (some fixed points, some flexible according to individual's condition)<br>[ ] full (each patient receives a unique and evolving diganosis and treatment)<br>[ ] Not reported		
	**Intervention**	**Control**	
Control Intervention		**sham/placebo (inactive laser to true acupuncture points)**	
Number of actual acupuncture points used per session	14		
Total number of treatments	18		
Frequency of treatment	3x/wk		
Duration of treatment	6 weeks		
Length of Follow-up Period	6 weeks		
Washout period in cross-over design	not applicable		
**B. Details of other acupuncture related intervention**	**Intervention**	**Control**	
Other acupuncture related interventions	[ ] needle acupuncture<br>[ ] acupressure<br>[ ] moxibustion<br>[ ] cupping<br>[ ] tuina<br>[ ] diet<br>[ ] qi gong<br>[ ] other<br>[x] none	[ ] needle acupuncture<br>[ ] acupressure<br>[ ] moxibustion<br>[ ] cupping<br>[ ] tuina<br>[ ] diet<br>[ ] qi gong<br>[ ] other<br>[x] none	
Location of other acupuncture related intervention	not applicable	not applicable	
Frequency of other acupuncture related intervention	not applicable	not applicable	
Rationale for use	[ ] based on previous studies<br>[ ] literature sources<br>[ ] Consensus methods<br>[ ] Other<br>[ ] Not reported<br>[x] Not applicable		
**C. Details of co-interventions (new)**	**Treatment**	**Control**	
Other interventions administered (new)	[ ] Language therapy<br>[ ] Behaviour therapy<br>[ ] Physical therapy<br>[x] Sensory integration - occupational therapy<br>[ ] Rich language environment<br>[ ] Medication<br>[ ] Other<br>*Check all that apply	[ ] Language therapy<br>[ ] Behaviour Therapy<br>[ ] Physical Therapy<br>[x] Sensory Integration - occuaptional therapy<br>[ ] Rich language environment<br>[ ] Medication<br>[ ] Other<br>*Check all that apply	
On-going treatments	no	no	
Frequency of co-intervention	not specified	not specified	
Rationale for use	not specified	not specified	
D. Instructions to practitioners	not specified		
Information and explanation to parents	yes	yes	
Parents signed informed consent	yes	yes	
	**Intervention**	**Control**	
E. Adverse event(s)	none	none	
**F. Laser Settings:**			
Type of laser used	RJ-Laser Laserpen Praxis type 130		
Power (mW)	40 mW		
Time of irradiation (seconds)	25 sec		
Frequency (Hz)	Nogier G 18688-Hz		
Dosage (J/cm² per acupuncture point)	1 Joule		
Wavelength (nm)	785 nm		

**Practitioner background**			
Type of practitioner	not specified		
Acupuncture licence/ laser acupuncture training	not specified		
Years of acupuncture and laser acupuncture practice	not specified		
Other relevant experience	not specified		

**Followup**			
	**Treatment**	**Control**	
Number of Dropouts	0	0	
Reason for Dropout	not applicable	not applicable	
Analysis type			

Intervention Group						Control Group					Relative Effects			
Name of Measurement Tool	Pre-Intervention Score	Post-Intervention Score	Mean Difference ± Standard Deviation	Confidence Interval (95%)	p-value	Pre Score	Post Score	Mean Difference ± Standard Deviation (Control)	Confidence Interval (95%) (Control)	p-value (control)	Mean ± SE	95% CI	statistically significant difference	p value
**WeeFIM**														
**Comprehension**			2.86 ± 0.86					0.95 ± 0.63					yes	**p<0.001**
**Expression**			1.73 ± 0.81					0.69 ± 0.55					yes	**p<0.001**
**Social Interaction**			2.86 ± 1.09					1.39 ± 0.78					yes	**p<0.001**
**Parental Report - Sensory Profile**			9.30 ± 9.39					7.74 ± 7.62					yes	**p<0.001**
**Childhood Autism Rating Scale (CARS)**														
**Preschool Language Scale**														
**Receptive language raw score (/10)**														
**Receptive language age**														
**Expressive language raw score**														
**Expressive language age**														
**Total language raw score**														
**Total language age**														
**Total language performance scaled score**														

Other outcomes reported but not used in this review			
biomarkers	☐		
mi-R levels	☐		
other	☐		

References to other trials:			
Did this report include any references to published reports of potentially eligible trials not already identified for this review			
If yes,			
Lead author			
Journal/Conference			
Year of publication			
Issue			
Did this report include any references to unpublished data from potentially eligible trials not already identified for this review?			
If yes,			
Lead author	Sujudi Y		
Title	Effects of Laserpuncture on Children with Autism		
Country of first author	Indonesia		
Journal/Conference/Academic Institution	Universitas Indonesia		
Year of publication	2004		
Issue	Not applicable		

Elsheikh et al. Data Extraction Form

Name of data extractors:	D Dong, L Xu		
Date of data extraction: DD/MM/YY	03/09/2024		
PROSPERO Study ID	CRD42024576887		

Characteristics of included studies			
Title	Efficacy of Laser Acupuncture for Children with Autism Spectrum Disorder: Clinical, Molecular, and Biochemical Study		
Lead Author (Last name, initials)	Elsheikh, M.S.		
Journal	Pediatric Neurology		
Year Published in print (latest publication will be used if there is publication errata).	2023		
Country of first author	Egypt		
Multicentre (Y/N)	No		
Setting(s)	research centre		
Trial registry ID			
Trial registration document/protocol available? If yes, append to study (URL, DOI, or PMID)			
Acupuncture paradigm (if described)	☑ TCM<br>☐ Western medical<br>☐ Japanese<br>☐ Korean<br>☐ Five Element<br>☐ Auricular<br>☐ Other<br>☐ Not specified		

Study Methods			
Study Design: Methods: description of study reads like controlled clinical trial "participants with ASD (n=30) were randomly divided into groups: Group I received LLLA therapy twice a week for 12 sessions and Group II did not receive LLLA sessions. Also, "This observational longitudinal study followed the checklist for Strengthening the Reporting of Observational Studies in Epidemiology."	☐ RCT<br>☐ Quasi RCT<br>☐ crossover<br>☐ Contolled Clinical Trial		
\\	Judgement	Support for Judgement (pg. number)	
Was random sequence generation used	Unclear		
Was allocation concealment used	Not reported		
Was there blinding of:			
a)participants	Not reported		
b)treating clinician	Not reported		
c)outcome assessor	Not reported		
d)statistician	Not reported		
e)data safety monitoring board	Not reported		
Source(s) of Funding	No funding		

Participant Characteristics			
Study's Participant Inclusion Criteria:	☑ Autism Diagnosis<br>☑ under 18 years old		
Study's Participant Exclusion Criteria	☐ psychiatric disorder<br>☑ other neurological condition<br>☑ neuropsychiatric disorder<br>☑ syndromic involvement<br>☑ Developmental Delay<br>☐ Psychotropic Drugs<br>☐ High Dose Corticosteroids<br>☑ medications<br>☐ Uncontrolled Epilepsy<br>☐ Medical emergency<br>☐ increased photoallergic response<br>☐ Exacerbation to chronic skin disorders<br>☑ Phoniatric treatment<br>☑ Motor delay<br>☐ Malignancy<br>☐ infections with fever<br>☐ increased photoallergic responses<br>☐ Uncooperative<br>☐ Other<br><br>*Check all that apply		
	Intervention	Control	Significant difference? (Comparability of groups at baseline) Do we want p-value
Sample Size	15	15	
Age range	5-12 years	5-12 years	
Mean Age ± S.D.	7.8± 2.8	8.5±2.7	No
Male:Female percentage	73.3% : 26.7%	66.7% : 33.3%	No
Severity of autism (CARS score range)	39.5±4.3	39.6±4.6	No
Speech scores (mean and range)			
comprehension	not applicable	not applicable	
expression	not applicable	notapplicable	
Social interaction	not applicable	not applicable	
Parental Report (Sensory Profile questionnaire)	not applicable	not applicable	

Trial Characteristics		
**A. Details of Laser Acupuncture Intervention**		
Names (or location if no standard name) of acupuncture points used	Check all that apply<br>☐ GB34 (Yanglingquan) - bilateral<br>☐ LR 3 (Taichong) - bilateral<br>☐ HT7 (Shenmen) - bilateral<br>☑ LI4 (Hegu) - bilateral<br>☑ Sp6 (Sanyinjiao) - bilateral<br>☑ ST36 (Zusanli) - bilateral<br>☑ GB20 (Fengchi) - bilateral<br>☑ GV20 (Baihui) - unilateral<br>☑ GV24 (Shenting) - unilateral<br>☑ GV26 (Shuigou) - unilateral<br>☑ Ex-HN1 (Sishencong) - set of 4 points<br>☑ Ex-HN3 (Yintang) - unilateral<br>☑ Scalp-Speech Area - a line on scalp<br>☐ Other	
Rationale for points	☑ based on previous studies<br>☐ iterature sources<br>☐ Consensus methods<br>☐ Other<br>☐ Not reported	
Extent to which treatment was individualised	☐ none (all patients receive the same treatment at all sessions)<br>☐ partial (some fixed points, some flexible according to individual's condition)<br>☐ full (each patient receives a unique and evolving diganosis and treatment)<br>☑ Not reported	
	**Intervention**	**Control**
Control Intervention		no treatment
Number of actual acupuncture points used per session	unclear	
Total number of treatments	12	
Frequency of treatment	2x/wk	
Duration of treatment	6 weeks	
Length of Follow-up Period -	6 weeks	
Washout period in cross-over design	not applicable	
**B. Details of other acupuncture related intervention**	**Intervention**	**Control**
Other acupuncture related interventions	☐ needle acupuncture<br>☐ acupressure<br>☐ moxibustion<br>☐ cupping<br>☐ tuina<br>☐ diet<br>☐ qi gong<br>☐ other<br>☑ none	☐ needle acupuncture<br>☐ acupressure<br>☐ moxibustion<br>☐ cupping<br>☐ tuina<br>☐ diet<br>☐ qi gong<br>☐ other<br>☑ none
Location of other acupuncture related intervention	notapplicable	notapplicable
Frequency of other acupuncture related intervention	not applicable	notapplicable
Rationale for use	☐ based on previous studies<br>☐ literature sources<br>☐ Consensus methods<br>☐ Other<br>☐ Not reported<br>☑ Not applicable	

C. Details of co-interventions (new)	Treatment	Control
Other interventions administered (new)	☐ Language therapy<br>☐ Behaviour therapy<br>☐ Physical therapy<br>☐ Sensory Integration - occupational therapy<br>☑ Rich language environment<br>☐ Medication<br>☐ Other<br>*Check all that apply	☐ Language therapy<br>☐ Behaviour Therapy<br>☐ Physical Therapy<br>☐ Sensory Integration - occuaptional therapy<br>☑ Rich language environment<br>☐ Medication<br>☐ Other<br>*Check all that apply
On-going treatments	no	no
Frequency of co-intervention	not specified	not specified
Rationale for use	notspecified	notspecified
D. Instructions to practitioners	notspecified	notspecified
Parents signed informed consent	yes	yes
Information and explanation to parents	yes	yes
	**Intervention**	**Control**
E. Adverse event(s)	none	none
**F. Laser Settings:**		
Type of laser used	Gallium aluminum arsenide laser	
Power (mW)	200 mW	
Time of irradiation (seconds)	12 sec.	
Frequency (Hz)	notspecified	
Dosage (J/cm² per acupuncture point)	3 J/cm²	
Wavelength (nm)	850 nm	

Practitioner background		
Type of practitioner	notspecified	
Acupuncture licence/ laser acupuncture training	notspecified	
Years of acupuncture and laser acupuncture practice	notspecified	
Other relevant experience.	notspecified	

Followup		
	**Treatment**	**Control**
Number of Dropouts	0	0
Reason for Dropout	notapplicable	notapplicable
Analysis type - no drop outs. Did not state ITT		

Intervention Group						Control Group					Relative Effect	
Name of Measurement Tool	Pre-Intervention Score	Post-Intervention Score	Mean Difference ± Standard Deviation	Confidence Interval (95%)	p-value	Pre Score	Post Score	Mean Difference ± Standard Deviation	Confidence Interval (95%) (Control)	p-value (control)	Mean ± SE	95% CI
**WeeFIM**												
**Comprehension**												
**Expression**												
**Social Interaction**												
**Parental Report - Sensory Profile**												
**Childhood Autism Rating Scale (CARS)**	39.5±4.3	**33.9 ± 3.9**	**(-) 5.6 ± 3.4**		< 0.001	39.6 ± 4.6	37.5 ± 5.2	(-) 2.1 ± 1.7		< 0.001	(-) 3.4 ± 1.0	**(-) 5.5 to (-) 1.4**
**Preschool Language Scale**												
**Receptive language raw score (/10)**	29.9 ± 10.4	31.5 ± 9.9	1.6 ± 1.4		0.001	31.0 ± 12.4	31.7 ± 12.2	0.7 ± 0.7		0.001	0.9 ± 0.4	**0.1 to 0.8**
**Receptive language age**	2.6 ± 1.2	2.8 ± 1.2	0.2 ± 0.2		< 0.001	2.6 ± 1.5	2.7 ± 1.5	0.1 ± 0.1		0.002	0.1 ± 0.0	**0.0 to 0.2**
**Expressive language raw score**	30.0 ± 11.7	31.7 ± 12.3	1.7 ± 1.5		0.001	32.7 ± 15.6	33.3 ± 15.8	0.7 ± 0.8		0.041	1.0 ± 0.4	**0.1 to 1.9**
**Expressive language age**	2.3 ± 1.2	2.5 ± 1.3	0.2 ± 0.2		0.001	2.6 ± 1.6	2.7 ± 1.6	0.1 ± 0.1		0.011	0.1 ± 0.0	**0.0 to 0.2**
**Total language raw score**	59.9 ± 21.3	63.2 ± 21.5	3.3 ± 2.4		<0.001	63.7 ± 22.7	65.0 ± 22.9	1.3 ± 1.2		0.001	2.1 ± 0.7	**0.6 to 3.5**
**Total language age**	2.4 ± 1.2	2.6 ± 1.2	0.2 ± 0.1		<0.001	2.6 ± 1.2	2.7 ± 1.2	0.1 ± 0.1		0.002	0.1 ± 0.0	**0.0 to 0.2**
**Total language performance scaled score**	52.8 ± 3.4	54.1 ± 4.2	1.3 ± 1.3		0.002	53.3 ± 2.5	53.7 ± 2.8	0.5 ± 0.6		0.014	0.9 ± 0.4	**0.1 to 1.7**

Other outcomes reported but not used in this review			
biomarkers	☑		
mi-Rlevels	☑		
other	☐		

References to other trials:			
Did this report include any references to published reports of potentially eligible trials not already identified for this review			
If yes,			
Lead author			
Journal/Conference			
Year of publication			
Issue			
Did this report include any references to unpublished data from potentially eligible trials not already identified for this review?			
If yes,			
Lead author	Sujudi Y		
Title	Effects of Laserpuncture on Children with Autism		
Country of first author	Indonesia		
Journal/Conference/Academic Institution	Universitas Indonesia		
Year of publication	2004		
Issue	Not applicable		

Appendix 4. PRISMA-A Checklist

PRISMA-A checklist

Subjects	PRISMA for Acupuncture
Title	
**Title**	1* Identify the report as a systematic review, meta-analysis, or both; if applicable, state the specific type of acupuncture treatment, such as manual acupuncture or electroacupuncture.
Abstract	
**Structured summary**	2† Provide a structured summary including, as applicable: background; objectives; data sources; study eligibility criteria, participants, and interventions; study appraisal and synthesis methods; results limitations; conclusions and implications of key findings; systematic review registration number.
Introduction	
**Rationale**	3* Describe the rationale for what is already known about acupuncture for the target condition in the background; if applicable, state what is already known about the specific types of acupuncture to be studied, and describe whether there is any difference of the effects among different types of acupuncture.
**Objectives**	4† Provide an explicit statement of questions being addressed with reference to participants, interventions, comparisons, outcomes, and study design (PICOS)
Methods	
**Protocol and registration**	5† Indicate if a review protocol exists, if and where it can be accessed (e.g., web address), and, if available, provide registration information including registration number.
**Eligibility criteria**	6† Specify study characteristics (e.g., PICOS, length of follow-up) and report characteristics (e.g., years considered, language, publication status) used as criteria for eligibility, giving rationale.<br>6a.1‡ Describe the diagnostic criteria of the target condition in Western medicine.<br>6a.2‡ If applicable, describe the diagnostic criteria in terms of Traditional Medicine, such as Traditional Chinese Medicine.<br>6b‡ Describe the types of acupuncture to be included, such as traditional acupuncture, electroacupuncture, or fire acupuncture.

	6c‡ If applicable, report measures for therapeutic effects using the terminology of either traditional medicine (e.g. syndrome score for syndrome remission) or Western medicine (e.g. pain intensity).
**Information sources**	7* Describe all sources of information (e.g., databases with dates of coverage, contact with study authors to identify additional studies) in the search, and report the date of the last search. If applicable, report the databases or complementary search methods for acupuncture or traditional medicine.
**Search**	8* Present full electronic search strategy for at least one commonly used database (e.g. MEDLINE), including any limits used, such that it could be repeated. If applicable, include the full search strategy for at least a Western and a traditional medicine database for each systematic review where both were used.
**Study selection**	9† State the process for selecting studies (i.e., screening, eligibility, included in systematic review, and, if applicable, included in the meta-analysis).
**Data collection process**	10† Describe method of data extraction from reports (e.g., piloted forms, independently, in duplicate) and any processes for obtaining and confirming data from investigators.
**Data items**	11* List and define all variables for which data were sought (e.g., PICOS, funding sources) and any assumptions and simplifications made; describe data items about details of acupuncture interventions and controls (**e.g.,** sham acupuncture) referring to TIDieR when applicable.
**Risk of bias in individual studies**	12† Describe methods used for assessing risk of bias of individual studies (including specification of whether this was done at the study or outcome level), and how this information is to be used in any data synthesis.
**Summary measures**	13† State the principal summary measures (e.g., risk ratio, difference in means).
**Synthesis of results**	14† Describe the methods of handling data and combining results of studies, if done, including measures of consistency (e.g., I^2) for each meta-analysis.
**Risk of bias across studies**	15† Specify any assessment of risk of bias that may affect the cumulative evidence (e.g., publication bias, selective reporting within studies).
**Additional analyses**	16† Describe methods of additional analyses (e.g., sensitivity or subgroup analyses, meta-regression), if done, indicating which were pre-specified.
Results	
